# Research on the vessel passing capacity of the Changshan waterway

**DOI:** 10.1371/journal.pone.0355503

**Published:** 2026-08-06

**Authors:** Rui Sun, Lvzhen Ren, Wen Ma, Luling Zeng

**Affiliations:** 1 Navigation College, Jimei University, Xiamen, China; 2 College of Marine Culture and Law, Jimei University, Xiamen, China; Sabancı Üniversitesi, TÜRKIYE

## Abstract

The Changshan Waterway is a key passage connecting the Bohai Sea and the Yellow Sea in northern China, where complex navigational conditions and heavy vessel traffic place increasing pressure on navigational safety and operational efficiency. To quantitatively assess the throughput capacity of this waterway, this study analyzes vessel traffic characteristics using Automatic Identification System (AIS) data and develops a simulation-based capacity evaluation model implemented in AnyLogic. The results indicate that the current theoretical annual throughput capacity of the Changshan Waterway is approximately 248,000 vessels, with an overall capacity saturation level of 66.4%, approaching the critical operating threshold. On this basis, optimization strategies focusing on vessel speed regulation and longitudinal spacing management are proposed to further enhance the throughput capacity of the waterway.

## 1. Introduction

Under conditions of high traffic demand and constrained navigation space, vessel traffic organization and channel capacity assessment have long been core concerns in maritime traffic engineering. With the continuous increase in transport tasks handled by coastal ports and major waterways, the importance of traffic separation schemes (TSS) in ensuring navigational safety and operational efficiency has become increasingly prominent. For narrow or semi-enclosed waterways implementing TSS, constraints in channel geometry, mandatory directional segregation, and highly heterogeneous vessel compositions overlap. This makes the traffic operation process show obvious nonlinear characteristics. The growth of traffic demand not only changes vessel speeds and encounter frequencies but may also induce longitudinal queuing and congestion propagation in local waters, significantly affecting the overall passing capacity of the waterway. In this context, how to conduct a reasonable and operable assessment of the capacity of complex TSS waterways at the engineering level has become a key technical issue in channel planning and traffic management.

Simulation technology has become an important tool for solving complex system problems in traffic engineering due to its high precision, strong dynamics, and flexibility. Its application in road and pedestrian traffic is relatively mature; studies such as subway station design optimization and high-density crowd evacuation simulation have established a complete theoretical system [1–3]. With the increasing complexity of water transport systems, simulation technology has been gradually extended and deepened in the maritime traffic field. Existing research mainly focuses on port channel capacity assessment, logistics system scheduling optimization, and microscopic traffic flow mechanism analysis.

Regarding service level assessment for port waters and approach channels, existing studies mostly use discrete event simulation or multi-agent technology combined with specific operation constraints. Wang et al. [4] focused on the main channel of Jinzhou Port and analyzed the nonlinear impact of increasing large vessel proportions on the overall service level. Building on this, Wang et al. [5] considered multiple constraints in Ningbo-Zhoushan Port to build a simulation model for ultra-large vessels, proposing measures to reduce waiting time by quantifying differences in vessel delays. For navigation risks of specific cargo types, Li et al. [6] simulated the operation process of LNG ships in Tianjin Port, revealing the impact on average waiting and queuing times in the port area. Similarly, Han et al. [7] utilized a multi-agent approach to model ships entering and leaving ports in the Qiongzhou Strait, while Han et al. [8] combined ship car-following models with queuing theory to verify channel capacity and service levels under different safety settings. In addition, for congestion problems at key inland nodes, Zhang et al. [9] combined traffic flow simulation with congestion models to propose methods for optimizing transport capacity structure, providing solutions to relieve traffic pressure at the Three Gorges Dam waters.

Based on capacity assessment, many scholars have devoted efforts to using simulation to solve complex port logistics scheduling and resource allocation problems to improve overall system efficiency. Targeting different terminal operation characteristics, Nezar et al. [10] built a distributed simulation model and verified its effectiveness in assessing unloading capacity at Narvik Port. Tian et al. [11] focused on container ship scheduling in Qinzhou Port, while Fan [12] simulated inland container and truck scheduling optimization; both studies demonstrated how simulation can reduce waiting times and improve resource occupancy. To solve multi-variable coupling problems in resource allocation, Chatterjee et al. [13] combined AI and machine learning to optimize quay crane allocation strategies, while Ramirez-Polo et al. [14] used black-box simulation combined with optimization algorithms to solve complex berth allocation problems (BAP), significantly improving terminal performance. Some studies also explored the synergistic effects of external environment and operation strategies. For example, Wang et al. [15] introduced weather variables into terminal simulation to analyze the correlation between meteorological factors and container arrival times; Mathias et al. [16] used data-driven strategies to optimize the trajectories of RTG cranes at Hakata Port; Shao et al. [17] established a truck appointment mechanism model to effectively alleviate low turnover efficiency caused by truck quantity limits. At a more macro planning level, Bergeron et al. [18] and Guo et al. [19] applied simulation to the dual-level planning of port expansion and inland waterway capacity extension, while Eskafi et al. [20] used Bayesian estimation to forecast port throughput trends. Minh et al. [21] and Santoso et al. [22] further discussed the balance mechanism between port size, cargo handling rates, and transport costs from the perspectives of cost strategy and lean operations.

With the popularity of Automatic Identification System (AIS) data and the development of intelligent algorithms, the research perspective has gradually shifted from macro system operation to the mathematical description of microscopic traffic flow mechanisms and navigation behaviors. Targeting vessel movement characteristics in complex environments, Xin et al. [23] built a microscopic traffic simulation model based on AIS data to analyze vessel behavior in the Xiazhi Gate channel. Qi et al. [24] proposed a spatiotemporal discretization traffic flow model based on the “standard ship” concept, effectively solving the difficulty of determining safety distances and collision avoidance timing in simulation. Liu et al. [25] combined data-driven speed control strategies to develop a port channel simulation model based on Cellular Automata (CA), providing new methods for assessing transport efficiency. In terms of navigation safety and collision avoidance decisions, Choi et al. [26] used Monte Carlo simulation to quantitatively assess the performance differences of WVO and its hybrid algorithms in port congestion areas. Potočnik et al. [27] built a model predictive control framework integrating chart path planning to simulate the dynamic obstacle avoidance process of autonomous ships in coastal ports. Additionally, regarding the impact of special water environments, Chillcce et al. [28] used hydrodynamic equations to build numerical simulation models, accurately predicting vessel maneuvering performance in shallow waters; Jiang et al. [29] proposed a traffic flow saturation calculation platform based on environmental stress models; Vinke et al. [30] focused on the impact of climate change on channel resilience, simulating the navigation capacity of Dutch inland waterways under low-flow extreme climates to provide a basis for climate-adaptive planning.

In summary, simulation technology combined with AIS data mining has become a mainstream paradigm for ship traffic characteristic analysis and capacity assessment. However, looking at existing literature, most empirical studies focus on port waters or inland waterways where the environment is relatively closed and traffic organization is rigid. Research on outer sea TSS waterways, which feature high environmental openness, complex flow patterns, and significant mixing of commercial and fishing vessels, is relatively scarce. Unlike internal port waters, outer sea channels are more severely affected by the natural environment, and the traffic flow presents strong randomness and high heterogeneity. If empirical models and general parameters from ports or rivers are directly applied, it is often difficult to accurately reflect the real navigation bottlenecks.

In view of this, this paper selects the Changshan Waterway, a typical restricted outer sea water area, as an empirical object. It proposes a capacity assessment framework integrating AIS data mining and microscopic simulation. The study first employs the Levenberg-Marquardt (L-M) optimization algorithm to perform nonlinear curve fitting on ship arrival patterns, speed, and length distributions extracted from AIS. This is done to obtain high-precision characteristic parameters that can accurately describe the strong randomness of outer sea traffic flow. On this basis, a microscopic traffic flow model is built using the AnyLogic simulation platform, strictly following TSS rules and channel geometric constraints. By gradually loading traffic demand for stress testing, the evolution characteristics of the waterway operation state are analyzed to identify the passing capacity level under stable conditions. The results of this study aim to verify the engineering applicability of this method in complex outer sea environments and provide quantitative references for capacity assessment and management of similar strategic channels.

## 2. Study area and vessel traffic flow characteristics

This chapter defines the spatial scope and data basis of the study, providing clearly specified inputs for subsequent vessels traffic flow parameter analysis and simulation modeling.

### 2.1. Study area

The Changshan Waterway is located in the southern part of the Bohai Strait and extends predominantly in an east–west direction. It serves as a major maritime corridor connecting the northern China coast with Northeast Asia and represents a critical access route for vessels entering and leaving the Bohai Bay. As a typical constrained waterway, the Changshan Waterway operates under a strict TSS, in which eastbound and westbound traffic lanes are clearly separated by a central separation zone. In addition, precautionary areas are established in several key locations to regulate vessel course alterations and encounter behaviors.

From the perspective of the maritime transport system, the Changshan Waterway functions as a prominent traffic hub with a highly complex navigational environment. On the one hand, the waterway accommodates a high concentration of trunk-route vessels, including large cargo ships, bulk carriers, and oil tankers operating between the Bohai Bay and offshore routes. On the other hand, regional passenger vessels, engineering service ships, and fishing vessels coexist in the same navigational space. This highly heterogeneous mix of vessel types results in significant variations in vessel size, maneuvering capability, and operating speed, which substantially increases the complexity of traffic organization within the waterway.

In terms of channel geometry, the Changshan Waterway exhibits typical characteristics of a routeing system. The westbound traffic lane is located between the separation zone and the northern boundary of the routeing system. Its width is approximately 2.5 nautical miles in the western section and 2.3 nautical miles in the eastern section, with a minimum width of 1 nautical mile and a total length of 14.7 nautical miles. The predominant traffic heading for westbound vessels is 282 degrees true. The eastbound traffic lane is located between the separation zone and the southern boundary of the routeing system, with widths of 2.2 nautical miles in the eastern section and 3.7 nautical miles in the western section. It has a minimum width of 1 nautical mile and a length of 9.8 nautical miles, and the main traffic heading is 102 degrees true. The eastern precautionary area is defined as a circular zone with a radius of 4 nautical miles centered at 37°58′.250 N, 121°02′.500 E, while the western precautionary area is defined by a radius of 5 nautical miles centered at 38°05′.000 N, 120°24′.600 E.

Due to geographical constraints and routeing arrangements, vessel movements in the Changshan Waterway are largely confined to predefined navigation routes, and lateral maneuvering space is relatively limited. Near channel turning points and within the precautionary areas at both the eastern and western ends, routes with different headings converge, forming several zones with intensive traffic interactions. In these areas, multi-directional vessel encounters occur frequently, which increases navigational complexity and places higher demands on vessel collision avoidance and decision-making.

### 2.2. AIS data source and preprocessing

AIS data provide high-frequency records of key vessel information, including Maritime Mobile Service Identity (MMSI), geographic position, speed, course, and vessel type and size. These data constitute a primary source for analyzing microscopic vessel traffic flow characteristics. In this study, AIS trajectory data of vessels navigating through the Changshan Waterway in March 2024 are selected as the analysis sample. This period corresponds to a typical spring operating season and reasonably represents traffic conditions under normal meteorological circumstances.

As raw AIS data are inevitably affected by signal drift, packet loss, and missing fields during wireless transmission and decoding, rigorous data preprocessing is required to ensure the reliability of subsequent statistical analysis. First, invalid records are removed, including those with abnormal MMSI identifiers, defined as non-nine-digit codes, position data outside the defined study area, or missing key motion parameters such as speed or course. Second, logical consistency checks based on vessel kinematic constraints are applied to filter out noise records, such as unrealistically high speeds, for example values exceeding 30 knots for conventional merchant vessels, or sudden position jumps occurring within a short time interval. Finally, discrete AIS records are reorganized into time-ordered trajectories according to vessel MMSI. For trajectory segments interrupted by temporary signal loss, linear interpolation is applied to restore continuity, resulting in a complete and continuous single-vessel trajectory dataset for subsequent analysis.

### 2.3. Traffic flow extraction based on the gate line method

To convert continuous spatiotemporal vessel trajectories into discrete traffic parameters suitable for statistical modeling, such as arrival times and cross-sectional traffic flow, a virtual gate line approach is adopted in this study. The gate line layout is designed by considering both the physical configuration of the Changshan Waterway and the dominant traffic directions. Four sets of virtual observation lines, denoted as L1 to L4, are positioned at key cross-sections of the waterway to capture vessel entry and exit behaviors.

The intersections between vessel trajectories and the gate lines are used to identify the precise time at which each vessel passes a given cross-section, thereby allowing the extraction of traffic parameters required for subsequent analysis and simulation input. The coordinates of the gate lines are listed in Table 1.

**Table 1:** Gate lines coordinates of target channel.

	Start point	End point
L_1_	(37°58.341′N,120°51.630E)	(38°0.078′N,120°41.274′)
L_2_	(37°59.510′N,120°51.904E)	(38°1.182′N,120°41.748′)
L_3_	(37°59.786′N,120°52.007E)	(38°1.539′N,120°41.769′)
L_4_	(38°00.727′N,120°52.223E)	(38°2.496′N,120°42.006′)

### 2.4. Fitting of vessel traffic flow characteristics

Based on the defined area and the processed AIS dataset, key parameters of vessel traffic flow in the Changshan Waterway are statistically analyzed and fitted with appropriate probability distributions. The resulting parameters are used as input for the subsequent AnyLogic simulation model.

**Fig 1:**
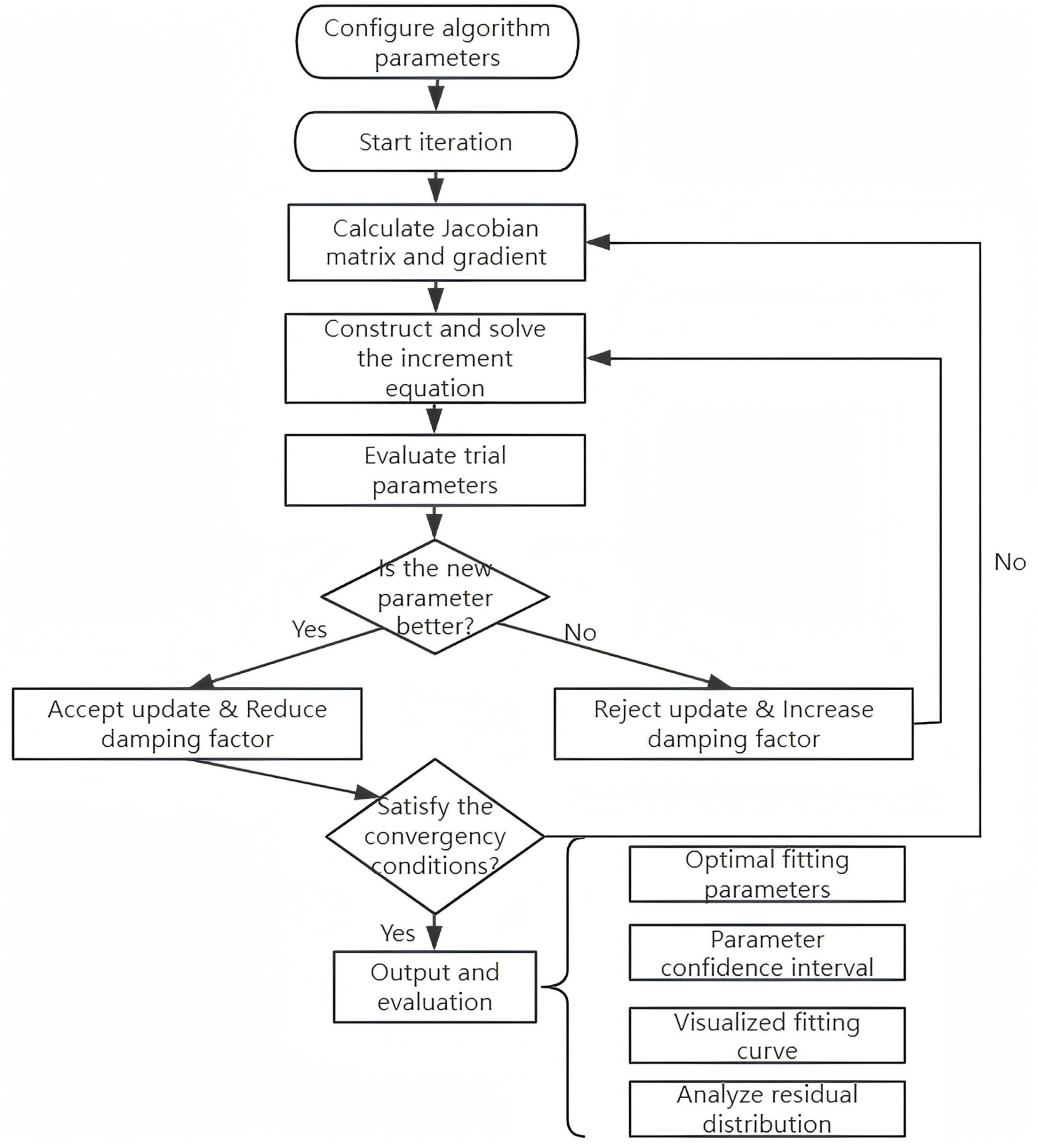
Workflow of traffic parameter fitting based on the L–M algorithm.

After data cleaning and traffic flow extraction using the gate line method, non-linear least squares fitting is applied to characterize the stochastic distributions of vessel traffic parameters. In this study, the L-M algorithm is adopted for parameter estimation. By combining the advantages of gradient descent and the Gauss-Newton approach, the L-M algorithm provides fast convergence and good numerical stability, making it suitable for AIS data-driven calibration of traffic flow parameters. The overall fitting procedure is illustrated in Fig 1.

#### 2.4.1. Distribution characteristics of vessel length.

Based on real-time monitoring data from the Changshan Waterway Vessel Traffic Service (VTS) system, vessel transit samples for March 2024 were collected using a stratified sampling approach. Key parameters, including vessel length and vessel type, were obtained from AIS records. After data cleaning and classification, a total of 7,152 valid vessel samples were retained, comprising 3,884 eastbound vessels and 3,268 westbound vessels.

**Fig 2:**
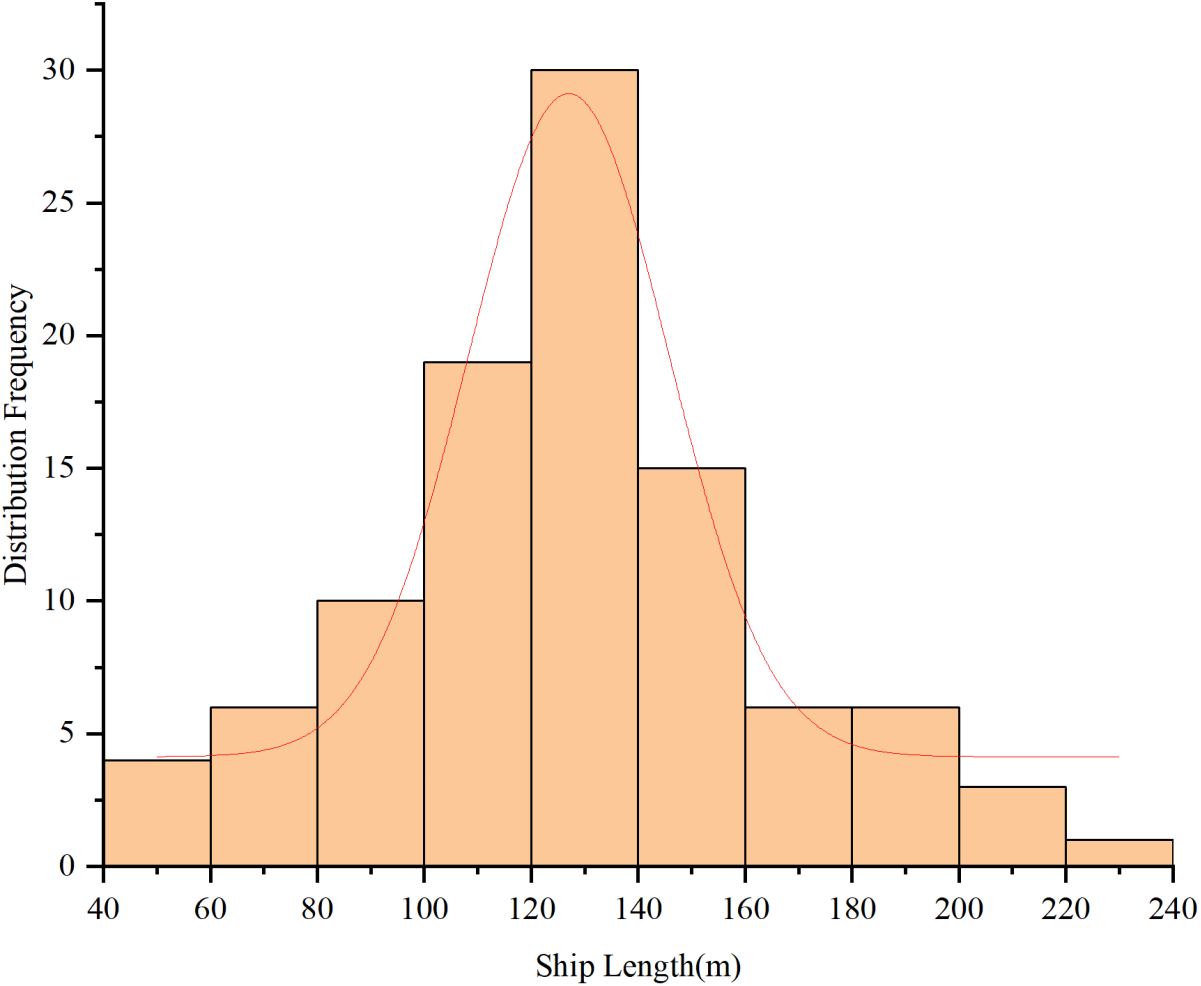
Length fitting curve for eastbound vessel.

**Fig 3:**
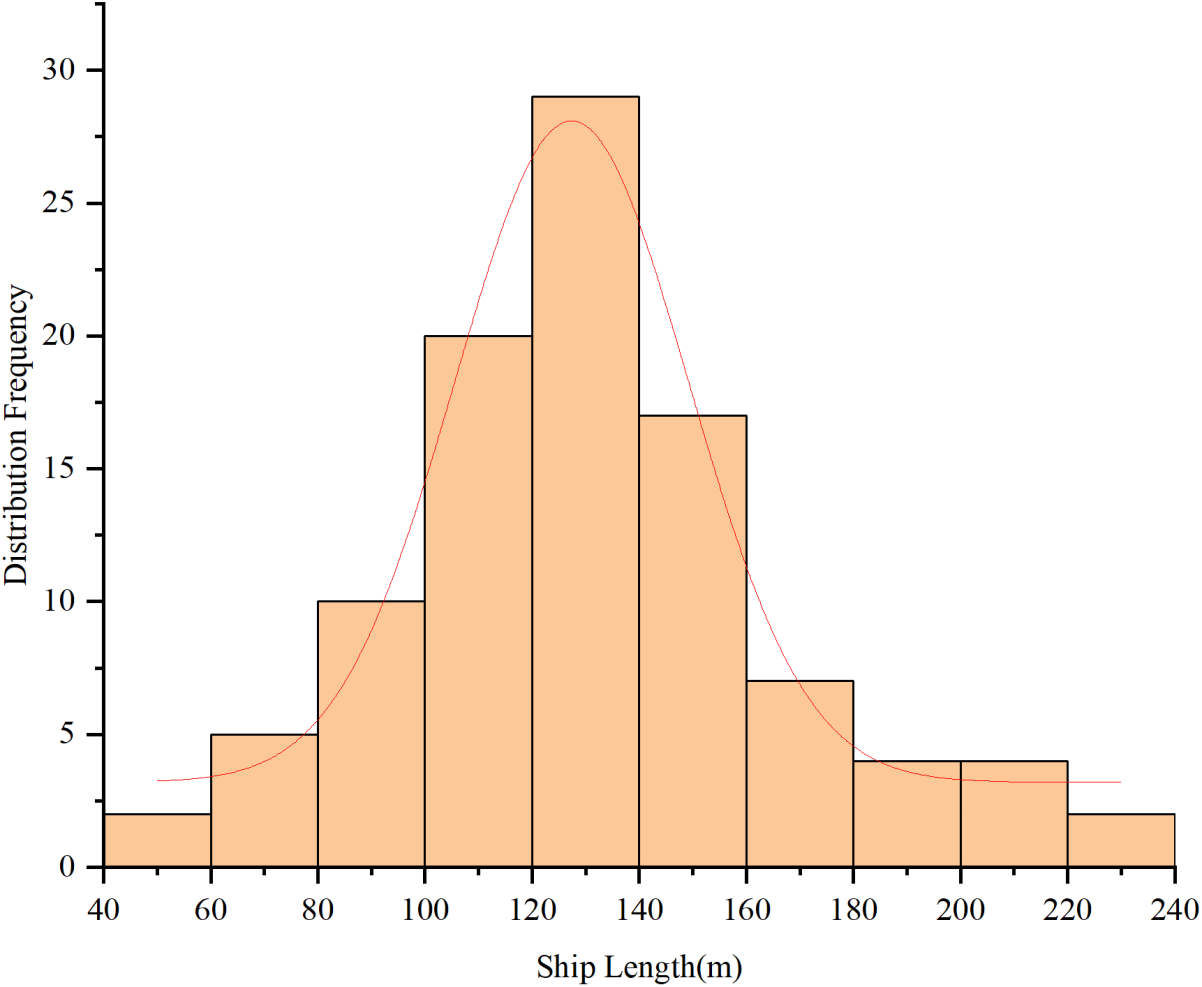
Length fitting curve for westbound vessel.

Nonlinear curve fitting was performed for vessel length distributions of eastbound and westbound traffic using the L-M algorithm with a Gaussian function. The fitted curves are shown in Figs 2 and 3, and the corresponding probability distribution functions are summarized in Tables 2 and 3.

**Table 2:** Probability distribution function of length for eastbound vessels.

Model	Gauss
Equation	$f(x)=y_{\text{0}}+\frac{A}{\sqrt{\text{2}\pi }\sigma }\text{exp}(-\frac{(x-\mu {)}^{\text{2}}}{\text{2}\sigma^{\text{2}}})$
$y_{\text{0}}$	3.78819 ± 0.9935
μ	128.55904 ± 1.78504
σ	38.37255 ± 4.05912
A	142.18076 ± 141.46513
*R* ^*2*^	0.96217
Adjusted *R*^*2*^	0.94326

**Table 3:** Probability distribution function of length for westbound vessels.

Model	Gauss
Equation	$f(x)=y_{\text{0}}+\frac{A}{\sqrt{\text{2}\pi }\sigma }\text{exp}(-\frac{(x-\mu {)}^{\text{2}}}{\text{2}\sigma^{\text{2}}})$
$y_{\text{0}}$	3.19934 ± 0.61064
μ	127.38432 ± 1.11666
σ	43.59713 ± 2.69224
A	136.15524 ± 92.94272
*R* ^*2*^	0.98734
Adjusted *R*^*2*^	0.98101

In the table, x is the independent variable, that is, ship length, in meters; $y_{\text{0}}$ is the baseline offset, representing the baseline height of the probability density function—that is, the background value of the probability density when the ship length deviates from the mean. The ideal value is 0, Here, it is slightly greater than 0 because noise in the data was accounted for during fitting; μ is the mean, representing the average ship length; σ is the standard deviation, measuring the dispersion of the ship length distribution; A is the amplitude coefficient, controlling the kurtosis or total area of the probability density curve. The parameters in Tables 6 and 7 have the same meanings.

The coefficient of determination (R²) is used to evaluate the goodness of fit of the regression models. For eastbound vessels, the initial R² value of the fitted curve was 0.96217, and it converged to 0.94326 after iterative optimization. The results indicate that the vessel length distribution for eastbound traffic is well approximated by a Gaussian distribution, with a mean length of 128.56 m and a standard deviation of 38.37 m.

For westbound vessels, the initial R² value was 0.98734 and converged to 0.98101 after optimization, also indicating good agreement with a Gaussian distribution. The corresponding mean vessel length is 127.38 m, with a standard deviation of 43.59 m.

#### 2.4.2. Vessel arrival patterns.

**Fig 4:**
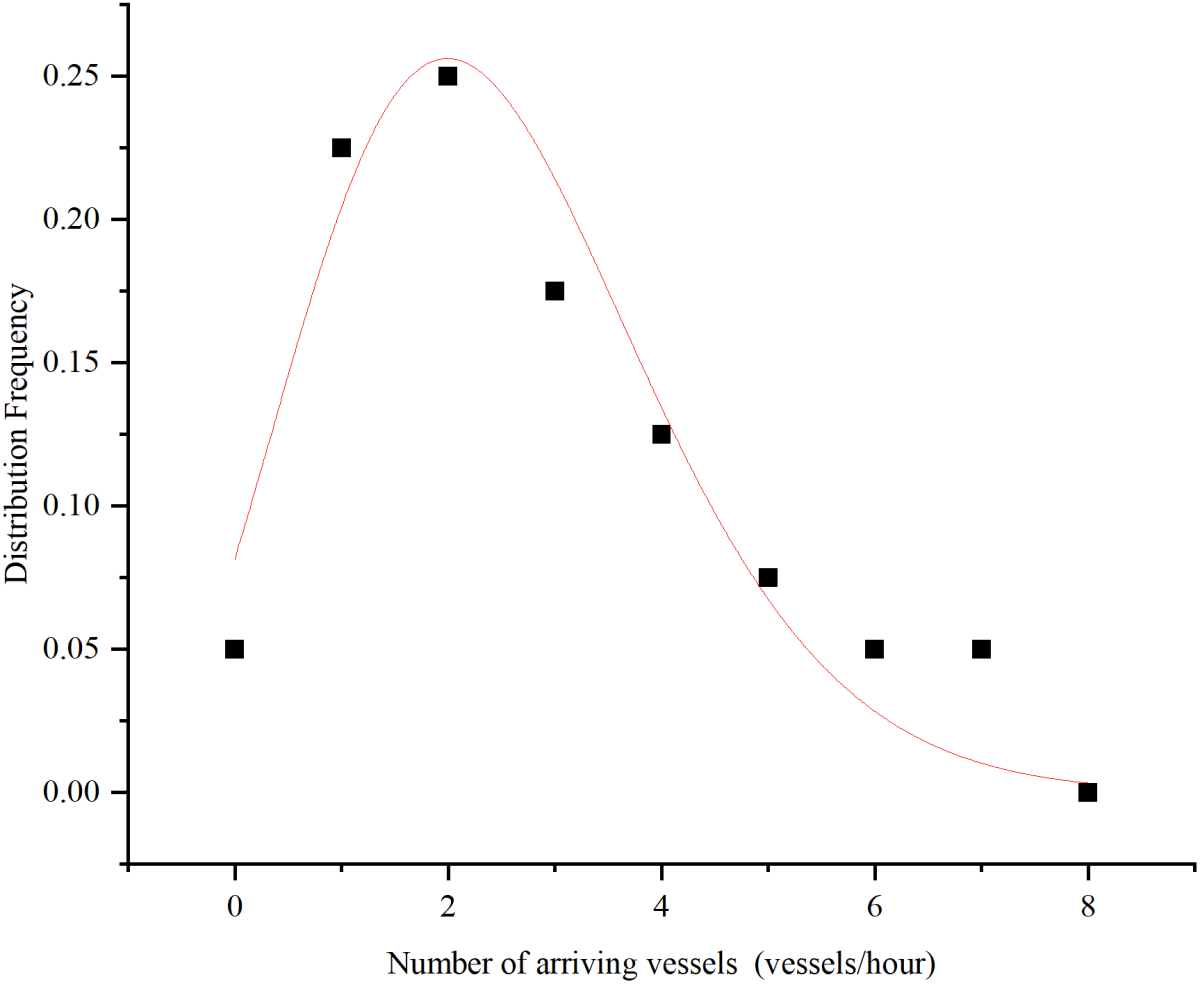
Distribution characteristics of eastbound vessels.

**Fig 5:**
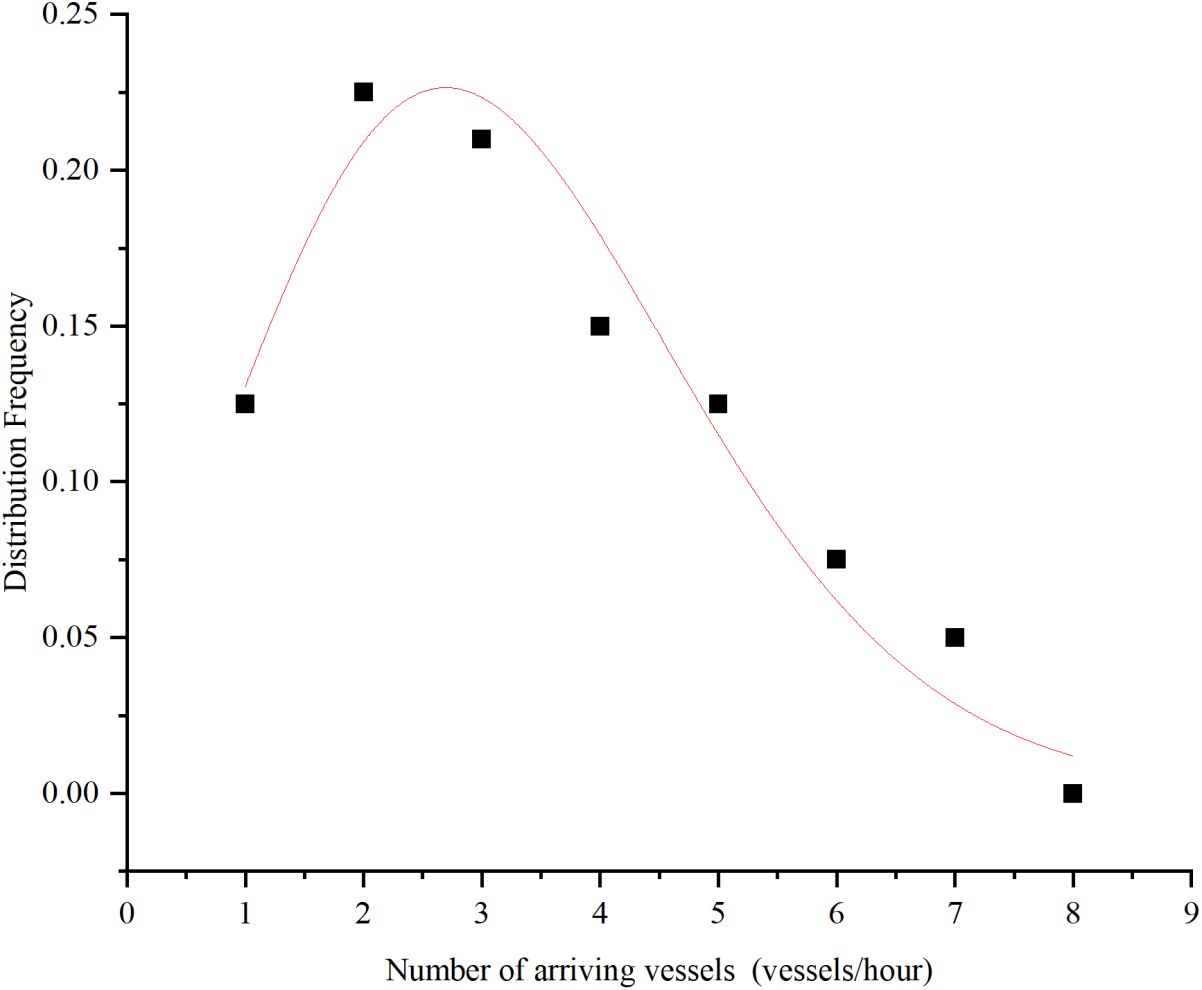
Distribution characteristics of westbound vessels.

Vessel arrival patterns describe the probabilistic characteristics of vessel arrivals at a specified location, either in terms of the inter-arrival time or the number of vessels arriving within a given time interval. Based on the extracted traffic flow data, nonlinear curve fitting was performed for the arrival processes of both eastbound and westbound vessels in the Changshan Waterway using the L-M algorithm in combination with a Poisson function. The fitting results are illustrated in Figs 4 and 5, and the corresponding fitted functions are summarized in Tables 4 and 5.

**Table 4:** Arrival pattern function table for eastbound vessels.

Model	Poisson
Equation	$P(X=k)=y_{\text{0}}+\frac{\lambda^{k}}{k!}e^{-\lambda },k=\text{0},\text{1},\ldots$
$y_{\text{0}}$	6.16882E-4 ± 0.00662
λ	2.67657 ± 0.10434
$R^{\text{2}}$	0.95852
Adjusted $R^{\text{2}}$	0.95259

**Table 5:** Arrival pattern function table for westbound vessels.

Model	Poisson
Equation	$P(X=k)=y_{\text{0}}+\frac{\lambda^{k}}{k!}e^{-\lambda },k=\text{0},\text{1},\ldots$
$y_{\text{0}}$	0.0034 ± 0.00645
λ	2.55528 ± 0.11577
*R* ^*2*^	0.95963
Adjusted *R*^*2*^	0.9529

In the table, k is the independent variable, representing the number of ships arriving per hour; P(X=k) is the dependent variable, representing the probability that exactly k ships arrive per hour; $y_{\text{0}}$ is the baseline offset; and λis the parameter of the Poisson distribution, representing the average number of ships arriving per unit of time.

**Table 6:** Probability distribution function table for eastbound vessels.

Model	Gauss
Equation	$f(x)=y_{\text{0}}+\frac{A}{\sqrt{\text{2}\pi }\sigma }\text{exp}(-\frac{(x-\mu {)}^{\text{2}}}{\text{2}\sigma^{\text{2}}})$
$y_{\text{0}}$	−0.8294 ± 2.07188
μ	10.15321 ± 0.07873
σ	2.9411 ± 0.31135
A	107.25963 ± 16.40882
*R* ^*2*^	0.98417
Adjusted *R*^*2*^	0.97231

**Table 7:** Probability distribution function table for westbound vessels.

Model	Gauss
Equation	$f(x)=y_{\text{0}}+\frac{A}{\sqrt{\text{2}\pi }\sigma }\text{exp}(-\frac{(x-\mu {)}^{\text{2}}}{\text{2}\sigma^{\text{2}}})$
$y_{\text{0}}$	0.1973 ± 3.02532
μ	9.9396 ± 0.12577
σ	2.89514 ± 0.48674
A	98.9023 ± 23.7626
*R* ^*2*^	0.96029
Adjusted *R*^*2*^	0.93051

For eastbound traffic, the initial R² value of the fitted curve was 0.95852 and converged to 0.95259 after iterative optimization. The results indicate that the arrival characteristics of eastbound vessels can be reasonably approximated by a Poisson distribution. In this case, the parameter λ represents the average number of vessel arrivals per hour, with an estimated value of 2.67.

Similarly, for westbound traffic, the fitted curve yielded an initial R² value of 0.95963 and converged to 0.95290 after optimization, also indicating good agreement with a Poisson distribution. The estimated λ value for westbound vessels is 2.55 vessels per hour.

#### 2.4.3. Vessel speed characteristics.

From a maritime traffic engineering perspective, vessel speed in a waterway is not considered solely at the individual ship level but is commonly represented by the average speed of all vessels passing through a given channel section, which is also referred to as the section-average speed. This measure reflects the overall operating state of the traffic flow under specific navigational and organizational conditions.

**Fig 6:**
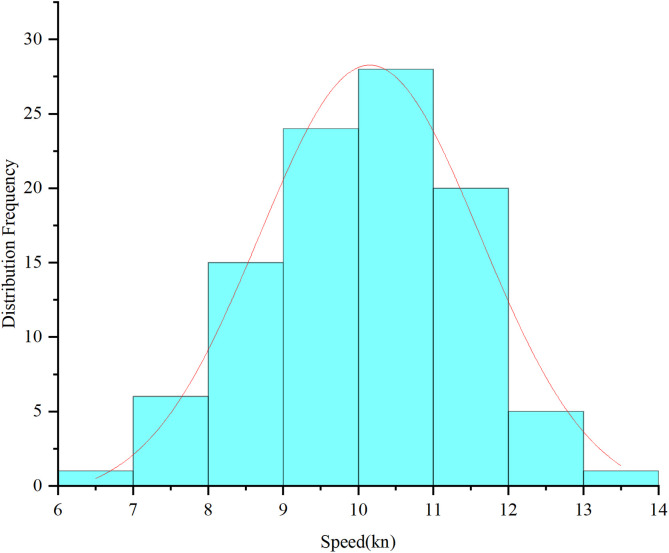
Speed distribution characteristics of eastbound vessels.

**Fig 7:**
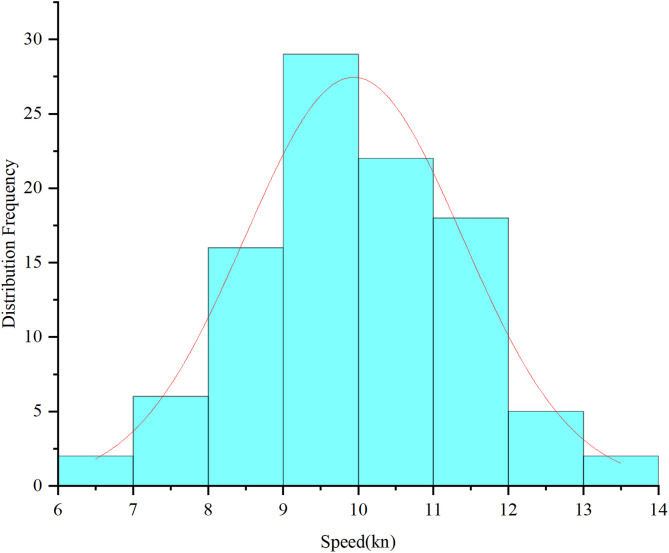
Speed distribution characteristics of westbound vessels.

Based on the AIS-derived speed data, nonlinear curve fitting was performed for the speed distributions of eastbound and westbound vessels in the Changshan Waterway using the L-M algorithm combined with a Gaussian function. The fitted results are shown in Figs 6 and 7, and the corresponding fitted functions are listed in Tables 6 and 7.

For eastbound traffic, the initial R² value of the fitted curve was 0.98417 and converged to 0.97231 after iterative optimization. The results indicate that the speed distribution of eastbound vessels can be reasonably approximated by a Gaussian distribution, showing good fitting performance. The estimated mean speed is 10.15 kn, with a standard deviation of 2.94 kn.

For westbound traffic, the fitting process yielded an initial R² value of 0.96029, which converged to 0.93051 after optimization. The speed distribution also exhibits a Gaussian-like pattern with satisfactory goodness of fit. The estimated mean speed is 9.94 kn, and the corresponding standard deviation is 2.89 kn.

#### 2.4.4. Longitudinal spacing between vessels.

The Haversine method is a core algorithm in spherical geometry, specifically designed to calculate the shortest great circle distance between any two points on the Earth’s surface. It is characterized by high accuracy and strong adaptability. Based on the differences in longitude and latitude between vessels, it uses the haversine function to correct spherical arc relationships, solves for the central angle, and combines this with the Earth’s radius to convert the result into actual surface mileage. The final output represents the true navigational distance between vessels on the Earth’s surface. To minimize errors, this paper employs the Haversine method to calculate the longitudinal distance between eastbound and westbound vessels in the Changshan Channel. Taking eastbound vessels as an example, the longitude and latitude coordinates of eastbound vessels obtained from AIS data were used to calculate and record the distances maintained between adjacent vessels during navigation using the Haversine method. After comprehensive analysis, the longitudinal spacing of vessels in the Changshan Channel was calculated. Table 8 shows the longitudinal spacing of some eastbound vessels.

**Table 8:** Data on vessels traveling eastward through the Changshan channel.

Ship	Longitude	Latitude	Length(m)	Distance(m)
Ship1	120.841200°E	37.981500°N	200	754
Ship2	120.835400°E	37.986200°N	159	
Ship3	120.835400°E	37.990800°N	110	927
Ship4	120.822500°E	37.983600°N	209	927
Ship5	120.816300°E	37.978200°N	96	943
Ship6	120.808700°E	37.973400°N	145	
……	……	……		
Ship99	120.714500°E	37.903500°N	115	902
Ship100	120.707100°E	37.898100°N	200	

After converting the distance between the stern and the bow into the ship's own length, the distance between the two ships is shown in Table 9.

**Table 9:** Table of vessel spacing expressed as a multiple of the vessel's length.

Ship	Longitude	Latitude	Length(m)	Distance(m)
Ship1	120.841200°E	37.981500°N	200	4.74
Ship2	120.835400°E	37.986200°N	159	
Ship3	120.835400°E	37.990800°N	110	4.43
Ship4	120.822500°E	37.983600°N	209	
Ship5	120.816300°E	37.978200°N	96	6.5
Ship6	120.808700°E	37.973400°N	145	
……	……	……		
Ship99	120.714500°E	37.903500°N	115	4.51
Ship100	120.707100°E	37.898100°N	200	

**Fig 8:**
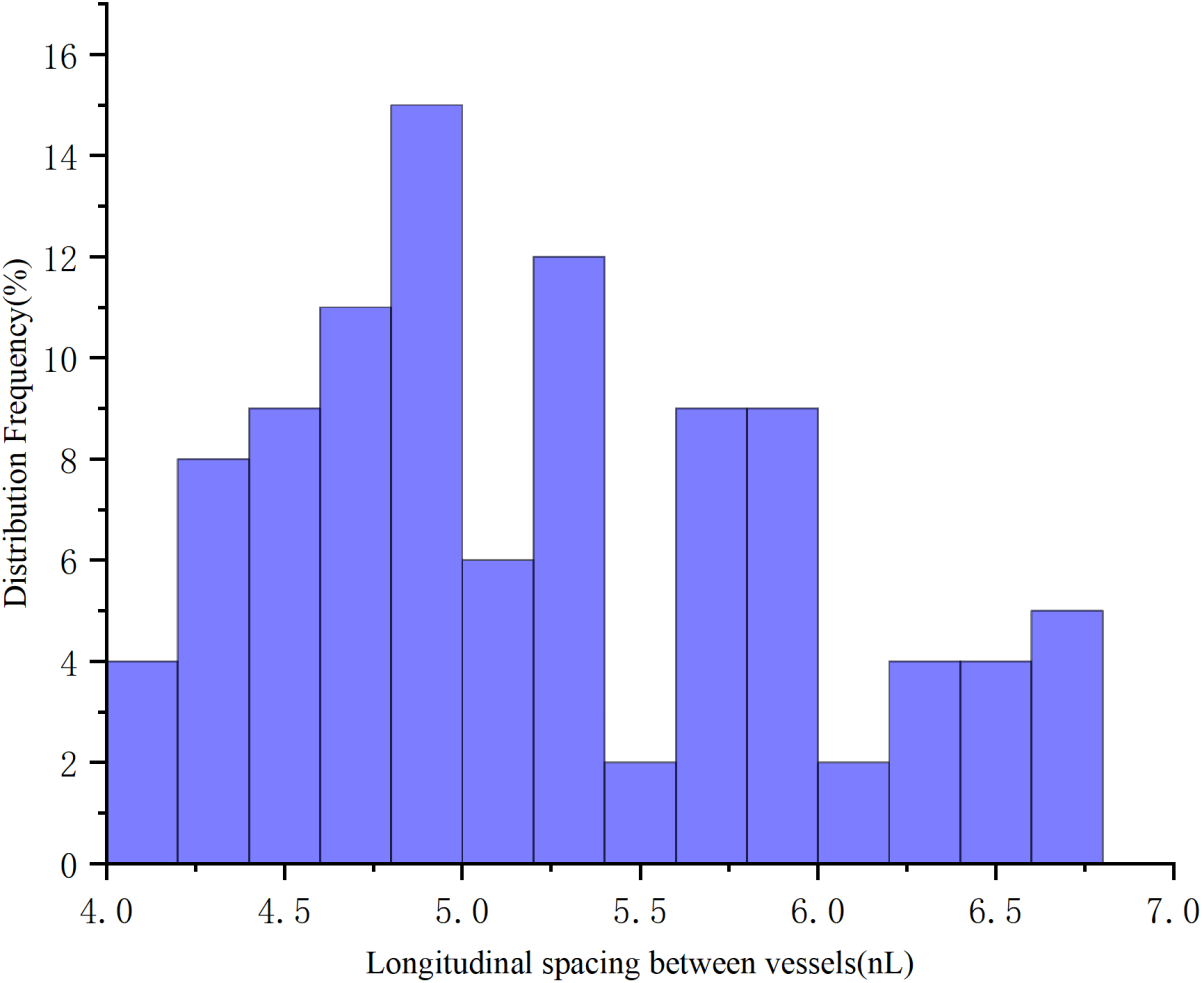
Histogram of ship longitudinal spacing.

Fig 8 shows a histogram of the longitudinal spacing between ships calculated from the table above.

Calculations show that the average longitudinal spacing for eastbound vessels in the Changshan Channel is 5.15 times the ship's length. By the same logic, the longitudinal spacing for westbound vessels is also approximately 5.15 times the ship’s length. Therefore, this paper adopts 5.15 times the ship’s length as the safe longitudinal distance to be maintained between vessels during the simulation. When the spacing between vessels deviates from this distance, the vessels will adjust their speed through the acceleration and deceleration modules within the simulation system to ensure a reasonable distance between the leading and trailing vessels.

## 3. Simulation model development and capacity analysis

### 3.1. Development of the vessel traffic flow simulation model

**Fig 9:**
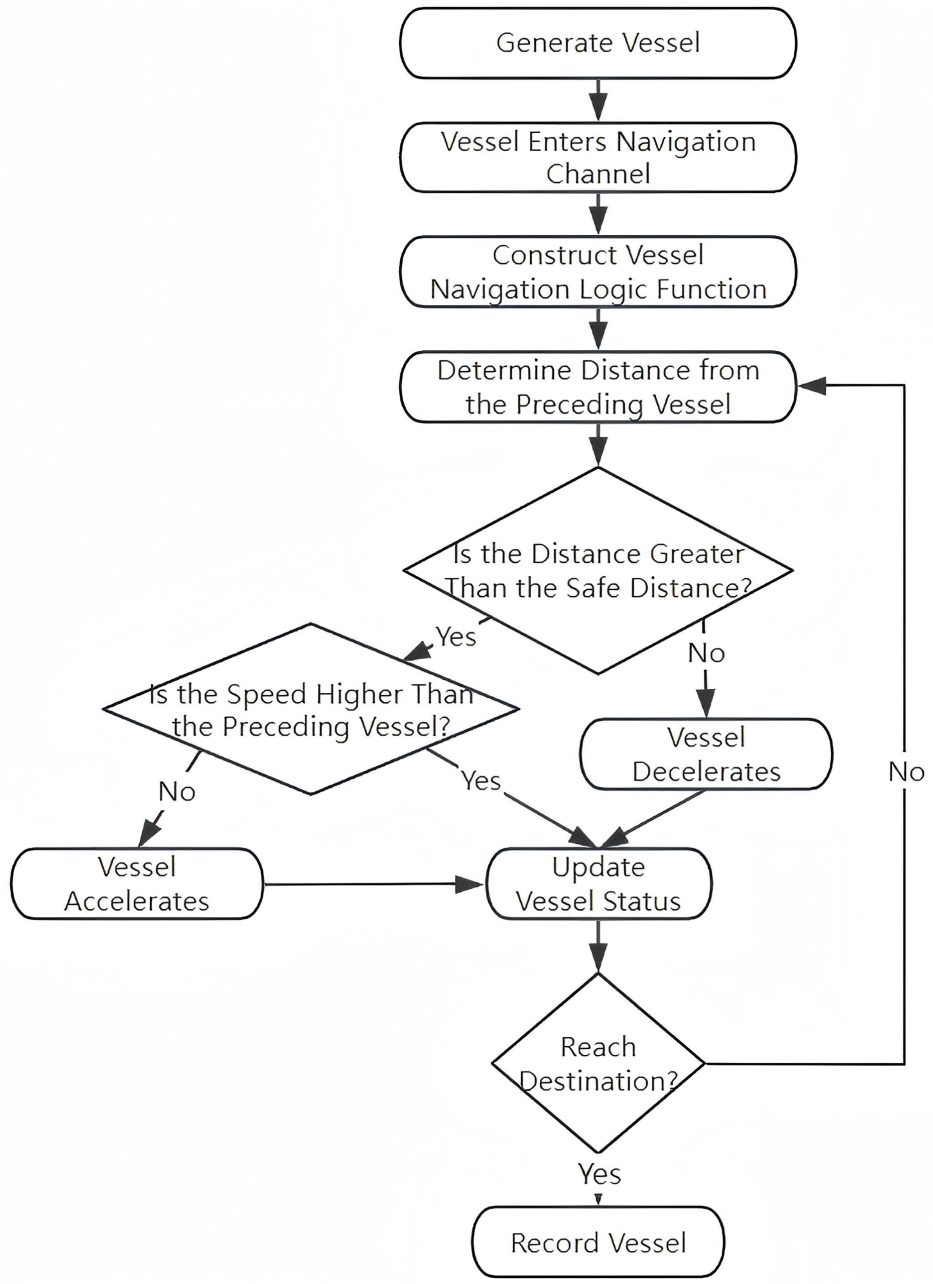
Simulation framework of the vessel traffic flow model.

Based on the analysis of vessel traffic flow characteristics in the Changshan Channel presented in Section 2.4, this paper develops a simulation model for vessel traffic flow in the Changshan Channel. The model consists of two main modules: vessel generation and vessel navigation. It enables dynamic simulation of vessel traffic flow in this waterway and uses the simulation results to estimate and analyze the capacity of the Changshan Channel. The overall operational workflow of the model is shown in Fig 9.

The vessel generation module produces vessels according to the fitted distributions obtained in Section 2, including vessel length distribution, arrival patterns, and speed characteristics. These statistically derived distributions ensure that the generated traffic demand reflects the observed characteristics of real vessel movements.

The vessel navigation logic incorporates a set of functional modules describing vessel behavior during transit, including acceleration and deceleration functions, environmental conditions represented by a simplified weather module, and distance-based judgment functions used to determine inter-vessel interactions and navigational responses.

Due to the inherent complexity of real-world maritime traffic systems and the limitations of available modeling techniques, it is neither feasible nor necessary to reproduce vessel traffic operations in a fully one-to-one manner through simulation. Moreover, an excessive level of detail may significantly increase model complexity and reduce computational efficiency without improving analytical value. Therefore, a set of reasonable modeling assumptions was adopted prior to model development.

It is assumed that vessels maintain stable maneuvering performance during navigation, with no sudden mechanical failures or exceptional incidents. Specifically, vessels are assumed to operate according to predefined speed profiles and acceleration or deceleration parameters, without experiencing abrupt speed changes or loss of control caused by equipment malfunction. In addition, vessel operators are assumed to comply strictly with established navigation rules and to exhibit adequate ship-handling skills and safety awareness throughout the voyage.

### 3.2. Validation of the simulation model

To evaluate the reliability of the simulation model, we compared daily simulated vessel counts with observed traffic data from March 2024 for both directions.

**Fig 10:**
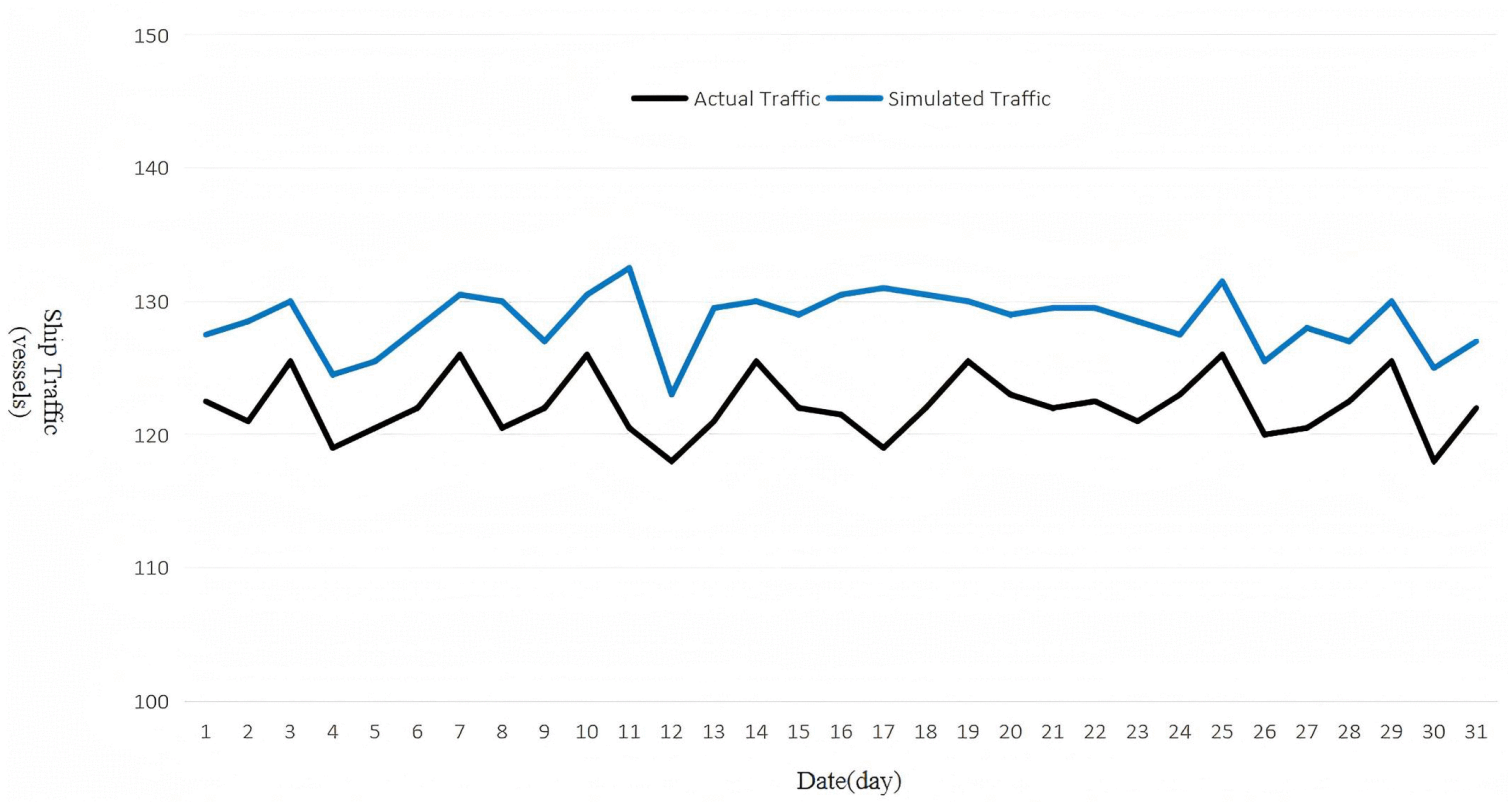
Comparison of eastbound vessel traffic for march.

**Fig 11:**
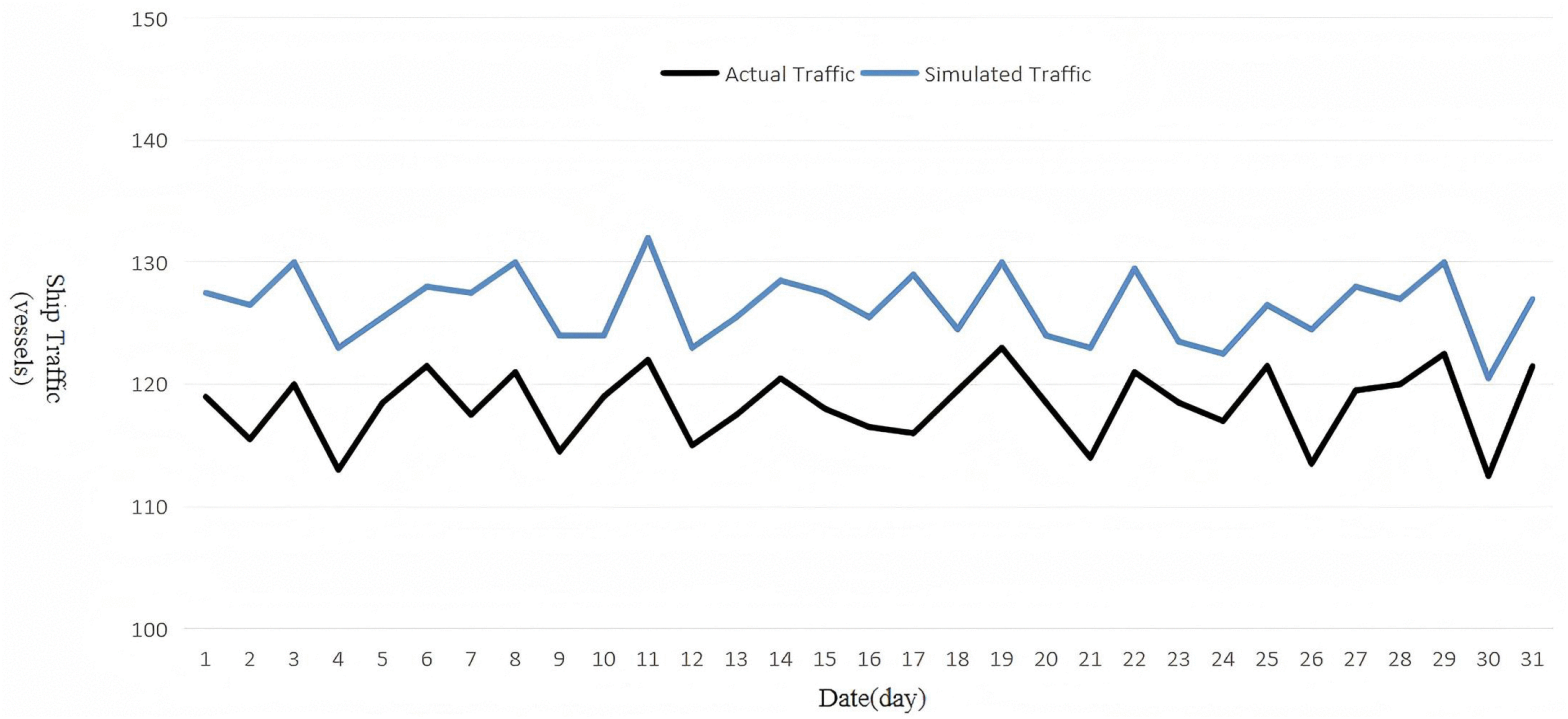
Comparison of eastbound vessel traffic for march.

The simulation model was executed under the calibrated traffic demand conditions, and the daily vessel volumes for the eastbound and westbound traffic lanes were recorded. The comparison between simulated and observed traffic flows is illustrated in Figs 10 and 11, while the corresponding quantitative errors are summarized in Table 10.

**Table 10:** Simulation results and actual flow error table.

	Actual Traffic (Vessels)	Simulated Traffic (Vessels)	Average Error (%)	Maximum Error (%)
Eastbound Vessel s	3786	3986	5.3	8.7
Westbound Vessels	3667	3917	6.8	9.5

As shown in Table 10, the total observed eastbound traffic volume in March was 3,786 vessels, whereas the simulation produced a total of 3,986 vessels. The average relative error was 5.3%, with a maximum error of 8.7%, which remained below 10%. For the westbound direction, the observed total traffic volume was 3,667 vessels, compared with 3,917 vessels obtained from simulation. The average error was 6.8%, and the maximum error was 9.5%, also within the 10% threshold.

Overall, the simulation outputs show a high degree of agreement with the observed traffic data in both traffic directions. This level of consistency indicates that the developed simulation model provides a reasonable representation of vessel traffic operations in the Changshan Waterway and is suitable for subsequent throughput capacity analysis.

### 3.3. Throughput capacity analysis of the Changshan waterway

#### 3.3.1. Throughput capacity estimation.

**Fig 12:**
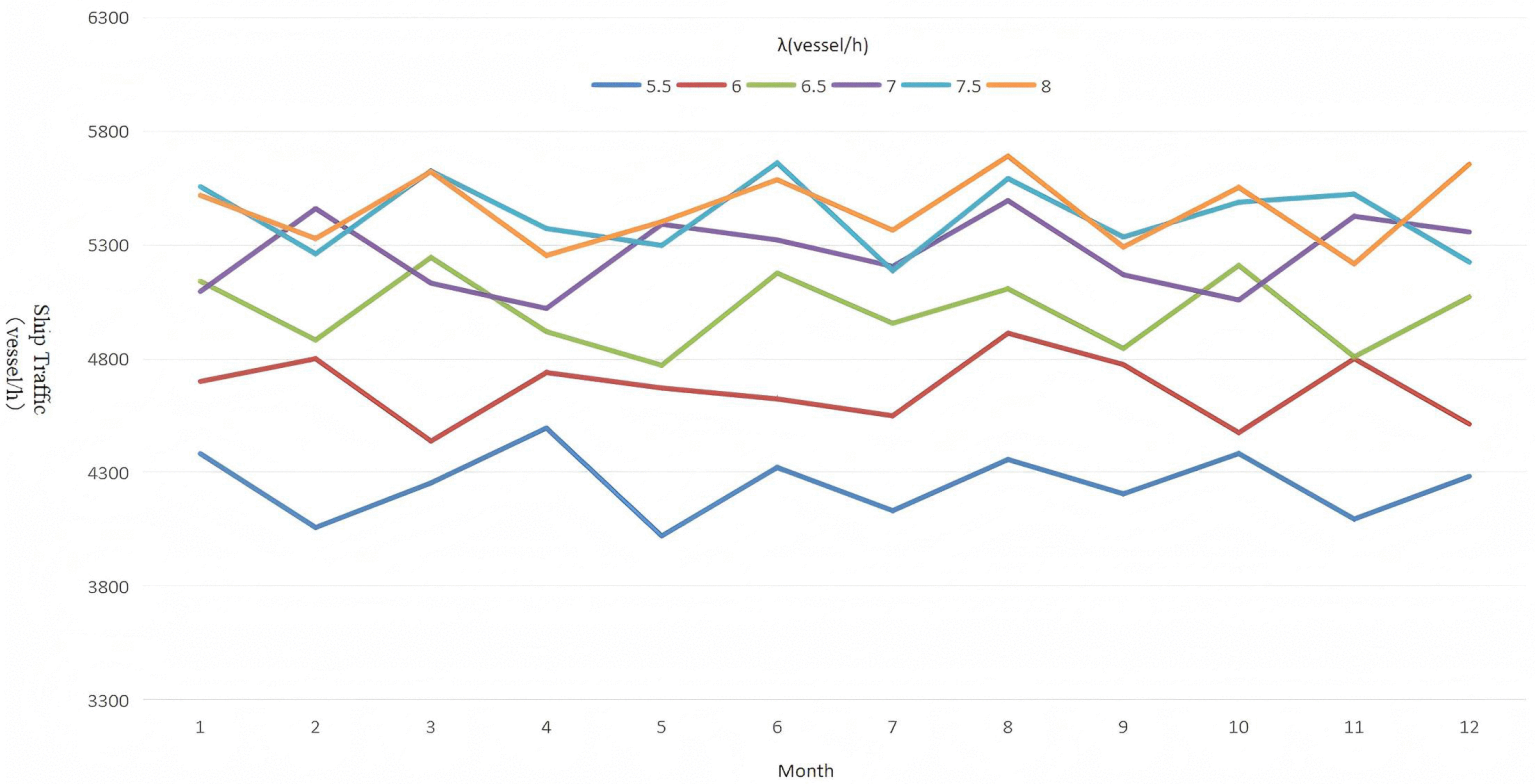
Simulated westbound traffic flow under different arrival rates (λ).

**Fig 13:**
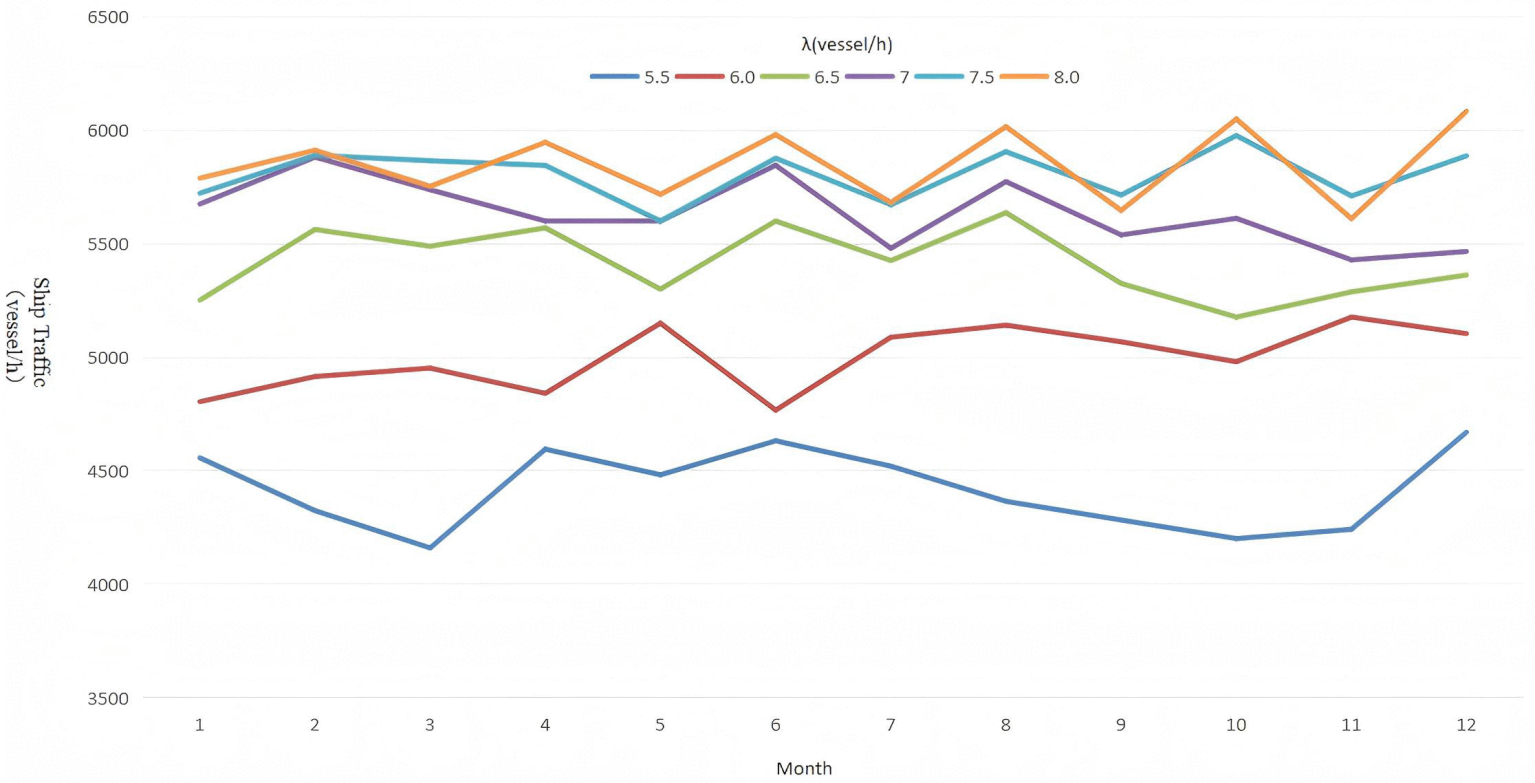
Simulated eastbound traffic flow under different arrival rates (λ).

Based on the statistical analysis of vessel arrival patterns, the average hourly arrival rates (λ) of vessels navigating the Changshan Waterway were estimated as 5.34 vessels/h for the eastbound channel and 5.10 vessels/h for the westbound channel. To investigate the relationship between traffic demand and waterway performance, six simulation scenarios were designed by progressively increasing the arrival rate λ to 5.5, 6.0, 6.5, 7.0, 7.5, and 8.0 vessels/h. The corresponding simulation outputs are presented in Figs 12 and 13.

As shown in Figs 12 and 13, for both westbound and eastbound traffic, the simulated vessel flow increases with λ at lower demand levels. When λ reaches 8.0 vessels/h, the resulting traffic flow becomes very close to that obtained under λ = 7.5 vessels/h, indicating that further increases in arrival demand no longer lead to a proportional growth in throughput. According to vessel traffic flow theory, this behavior suggests that the Changshan Waterway operates near saturation when λ = 8.0 vessels/h, and the corresponding traffic flow can be regarded as the practical throughput capacity of the waterway.

By aggregating the simulated vessel flows under λ = 8.0 vessels/h over all months, the annual throughput capacity of the westbound channel is estimated at 65,248 vessels, while that of the eastbound channel is approximately 70,132 vessels. The combined annual throughput capacity of the Changshan Waterway is therefore calculated as 135,380 vessels.

During the simulation process, vessels of different lengths are explicitly represented. If throughput capacity were calculated by simply counting each vessel as one equivalent unit, the resulting capacity estimate would be subject to noticeable bias. To better reflect actual operating conditions, it is necessary to account for differences in vessel size by introducing vessel equivalency factors. The vessel conversion coefficients adopted in this study follow the Chinese Ship Index Method, as listed in Table 11. The conversion of actual vessel flows into equivalent standard vessels is performed using Equation (1).

**Table 11:** Ship conversion factors for the China ship index method.

Length Overall(m)	Gross tonnage(t)	Conversion factor
≧246	≧60000	4
215 ~ 246	40000 ~ 60000	3
195 ~ 215	30000 ~ 40000	2.5
170 ~ 195	20000 ~ 30000	2.25
155 ~ 170	15000 ~ 20000	2
135 ~ 155	10000 ~ 15000	1.7
115 ~ 135	6000 ~ 10000	1.41
90 ~ 115	3000 ~ 6000	1.18
50 ~ 90	500 ~ 3000	1
30 ~ 50	100 ~ 500	0.5
≦30	≦100	0.25


$$\begin{array}{c} Q_{s}=Q*a \end{array}$$ (1)

In Equation (1), $Q_{s}$*Q_s_* denotes the converted actual vessel flow rate (vessels/n mile), Q represents the simulated vessel flow rate (vessels/n mile), and *a* is the vessel equivalency coefficient. Based on the length composition of different vessel types navigating the Changshan Waterway, the vessel equivalency coefficients for the eastbound and westbound channels are summarized in Tables 12 and 13.

**Table 12:** Conversion factors for eastbound vessels.

	Conversion factor	proportion	a
50m-90m	1	4.9	1.85
90m-150m	1.43	52.5	
150m-246m	2.44	42.2	
≥246m	4	0.4	

**Table 13:** Conversion factors for westbound vessels.

	Conversion factor	proportion	a
50m-90m	1	6.3	1.82
90m-150m	1.43	53.2	
150m-246m	2.44	40.1	
≥246m	4	0.4	

By applying the corresponding equivalency coefficients to the simulated traffic flows of the eastbound and westbound channels, the converted annual vessel flow is estimated to be 118,751 vessels for the westbound direction and 129,744 vessels for the eastbound direction. The combined annual vessel flow therefore reaches 248,495 vessels, which is taken as the throughput capacity of the Changshan Waterway.

#### 3.3.2. Channel saturation analysis.

Saturation analysis is an essential component of throughput capacity assessment, as it provides a direct measure of the relationship between existing traffic demand and the available channel capacity. Channel saturation is defined as the ratio of the actual traffic volume served under real operating conditions to the potential throughput capacity of the channel. The saturation index is generally expressed as a percentage, as shown in Equation (2).


$$\begin{array}{c} S=\frac{V}{C}*\text{1}\text{0}\text{0}\% \end{array}$$ (2)

According to commonly adopted standards in waterway traffic engineering, channel saturation is classified into six levels, each corresponding to a distinct service level and traffic flow condition. The relationship between channel saturation, service level, and traffic state is summarized in Table 14.

**Table 14:** Service levels and traffic flow conditions reflected by different channel saturation levels.

Waterway Saturation	Service Level	Traffic Flow Conditions
<40%	A	Smooth, no congestion
[40%−50%)	B	Stable, no congestion
[50%−70%)	C	Stable, no significant congestion
[70%−90%)	D	Unstable, minor congestion
[90%−100%)	E	Unstable, moderate congestion
>100%	F	Unstable, severe congestion

Based on the simulation results and vessel conversion factors, the eastbound channel of the Changshan Waterway exhibits a throughput capacity of 129,744 vessels per year. Its observed annual vessel count is 45,586, which corresponds to an equivalent traffic volume of 84,334 vessels per year after conversion, yielding a saturation level of 65.1%. This places the eastbound channel within Level C service conditions, characterized by stable operations and the absence of pronounced congestion.

Similarly, the westbound channel has an estimated throughput capacity of 118,751 vessels per year. The recorded annual traffic volume is 44,369 vessels, corresponding to an equivalent flow of 80,751 vessels per year. The resulting saturation level is 68.0%, which is slightly higher than that of the eastbound channel but remains within the Level C service range. At this level, traffic flow remains generally stable, although the margin to higher congestion states is limited.

When both traffic directions are considered together, the overall throughput capacity of the Changshan Waterway is estimated at 248,495 vessels per year. The total observed annual traffic volume is 89,955 vessels, corresponding to an equivalent flow of 165,085 vessels per year. This results in an overall saturation level of 66.4%, which is likewise classified as Level C service, as summarized in Table 15.

**Table 15:** Eastbound and westbound segments and comprehensive channel saturation.

	Actual Traffic	Through capability	Channel Saturation	Traffic Flow Conditions	Service Level
Eastbound	84334	129744	65.1%	Stable, no significant congestion	C
Westbound	80751	118751	68.0%	Stable, no significant congestion	C
Comprehensive Channel	165085	248495	66.4%	Stable, no significant congestion	C

As shown in Table 15, the saturation levels of the eastbound channel, westbound channel, and the integrated waterway are all approaching the upper boundary of the Level C range, which is commonly considered a critical threshold near 70 percent. At this stage, the traffic state may be described as quasi-congested, where small increases in traffic demand can lead to disproportionate declines in operational efficiency. With the continued growth of maritime transportation and regional economic activity, vessel traffic through the Changshan Waterway is expected to increase further. Once the saturation level exceeds 70 percent, the channel enters a transitional regime associated with increased congestion risk and reduced operational robustness.

From an engineering perspective, saturation levels below 70 percent indicate that the channel retains a certain degree of capacity redundancy, allowing for a balance between safety, efficiency, and operational cost. When saturation exceeds this threshold, the system enters a high-load operating state, in which safety risks, efficiency losses, and cost escalation tend to reinforce each other. As saturation continues to rise, particularly beyond 80 percent, these adverse effects may intensify rapidly and, in extreme cases, lead to prolonged operational disruptions.

In summary, the current saturation level of the Changshan Waterway is close to its critical threshold. Without effective intervention, continued growth in traffic demand is likely to adversely affect normal navigation conditions. As indicated by Equation (2), channel saturation may be reduced either by lowering traffic demand or by enhancing throughput capacity. Given the practical constraints on controlling vessel demand, this study focuses on strategies aimed at improving the throughput capacity of the Changshan Waterway in order to mitigate saturation growth.

### 3.4. Optimization strategies for throughput capacity improvement

#### 3.4.1. Reasonable planning of vessel sailing speed.

Sailing speed is a sensitive parameter affecting channel throughput capacity. To explore appropriate speed control strategies under constrained channel conditions, a sensitivity analysis was conducted by varying the average vessel speed while keeping other input parameters unchanged.

The throughput capacity obtained in Section 3.3 was based on a mean vessel arrival rate of λ = 8.0 vessels per hour. Under this condition, the average sailing speed followed a normal distribution with a mean value μ = 10.15 kn for eastbound vessels and μ = 9.94 kn for westbound vessels. In this subsection, the mean sailing speed μ for both traffic directions was adjusted to 10.2, 10.4, 10.6, 10.8, and 11.0 kn, respectively, and a series of simulation experiments were conducted to examine the variation in throughput capacity under different speed levels.

**Fig 14:**
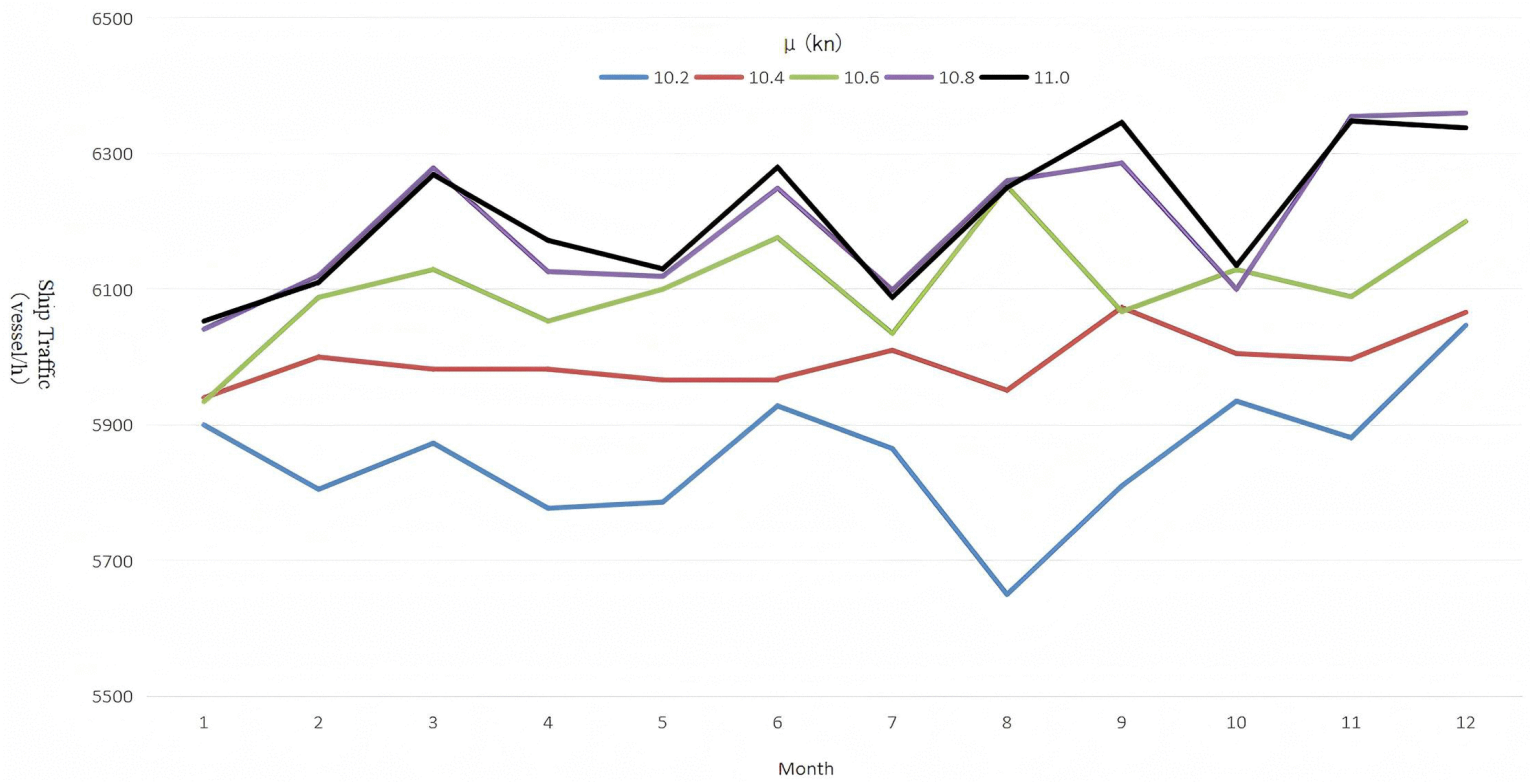
Changes in throughput capacity at different average speeds for the eastbound.

**Fig 15:**
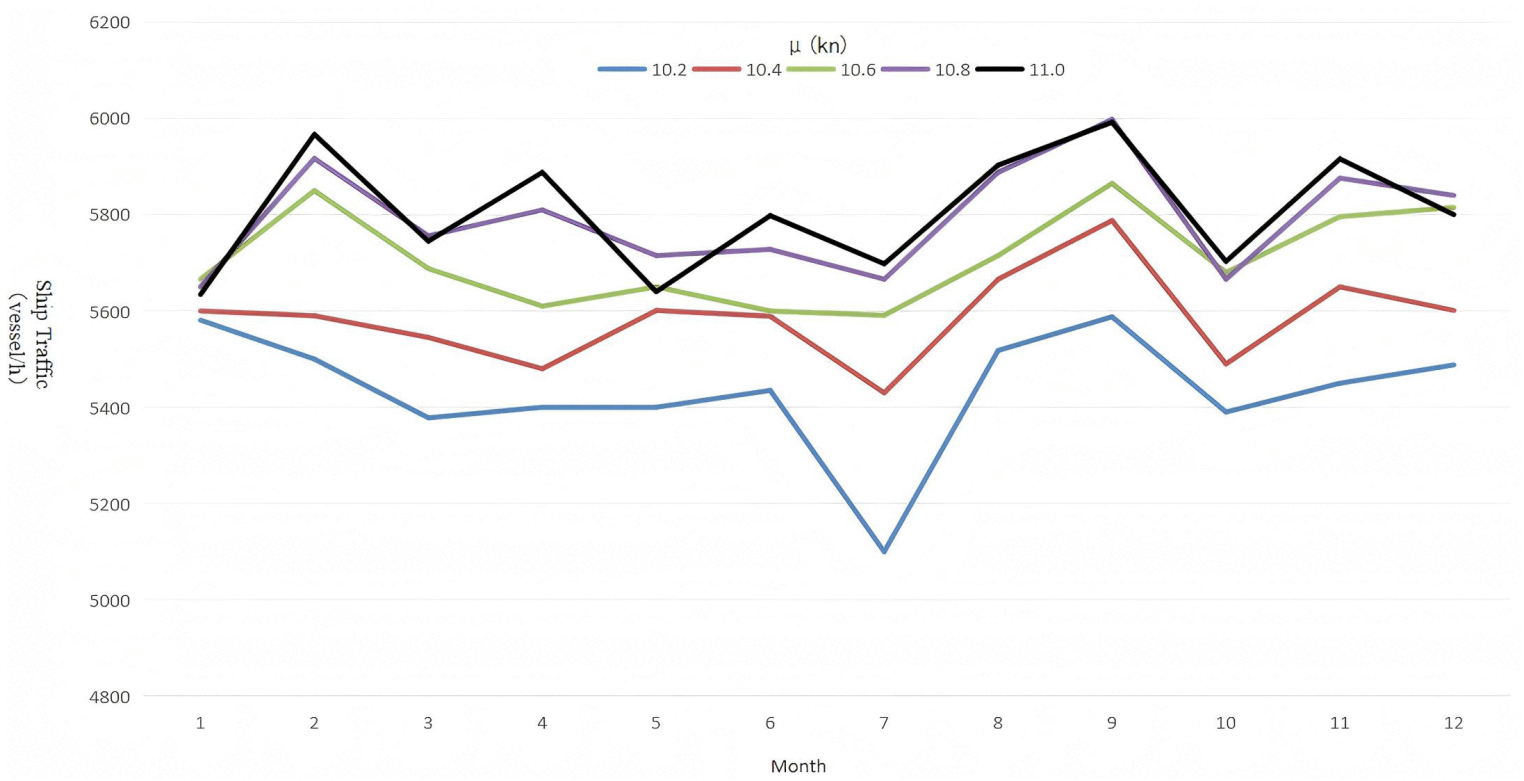
Changes in throughput capacity at different average speeds for the westbound.

The simulation results indicate that channel throughput capacity increases with rising vessel speed. However, the rate of increase gradually diminishes, reflecting a clear trend of diminishing marginal benefits. As shown in Figs 14 and 15, both eastbound and westbound channels exhibit continuous growth in throughput capacity as μ increases, although the growth rate slows noticeably at higher speed levels. When μ reaches 11.0 kn, the corresponding throughput capacity becomes very close to that obtained at μ = 10.8 kn, suggesting the presence of a critical speed threshold. Further increases in mean sailing speed beyond this point lead to a decline in throughput capacity. At μ = 11.0 kn, the throughput capacity of the eastbound channel reaches 74,159 vessels per year, representing an increase of 6.26 percent compared with the baseline capacity of 70,132 vessels per year at μ = 10.15 kn. Similarly, the westbound channel achieves a throughput capacity of 69,685 vessels per year, which is 6.81 percent higher than the baseline value of 65,248 vessels per year at μ = 9.94 kn.

These results indicate that moderate increases in vessel sailing speed can enhance navigation efficiency and improve channel throughput capacity. However, excessive speed growth does not lead to further capacity gains and may even reduce system performance. Based on the simulation outcomes, it is recommended that vessel speeds within the Changshan Waterway be maintained within the range of 10–11 kn. This range allows for stable vessel operations while effectively improving navigation efficiency and overall channel throughput capacity.

#### 3.4.2. Control of longitudinal vessel spacing.

Longitudinal safety spacing directly determines the spatial occupancy of a navigation channel. Similar to vessel speed, there exists a critical threshold for longitudinal spacing between vessels. When the spacing remains above this threshold, reducing the longitudinal distance leads to higher vessel density within a given channel segment and increases the number of vessels passing per unit time, thereby enhancing throughput capacity. However, when the spacing falls below the safety threshold, further reduction may increase the risk of incidents and consequently have a negative impact on channel performance.

**Fig 16:**
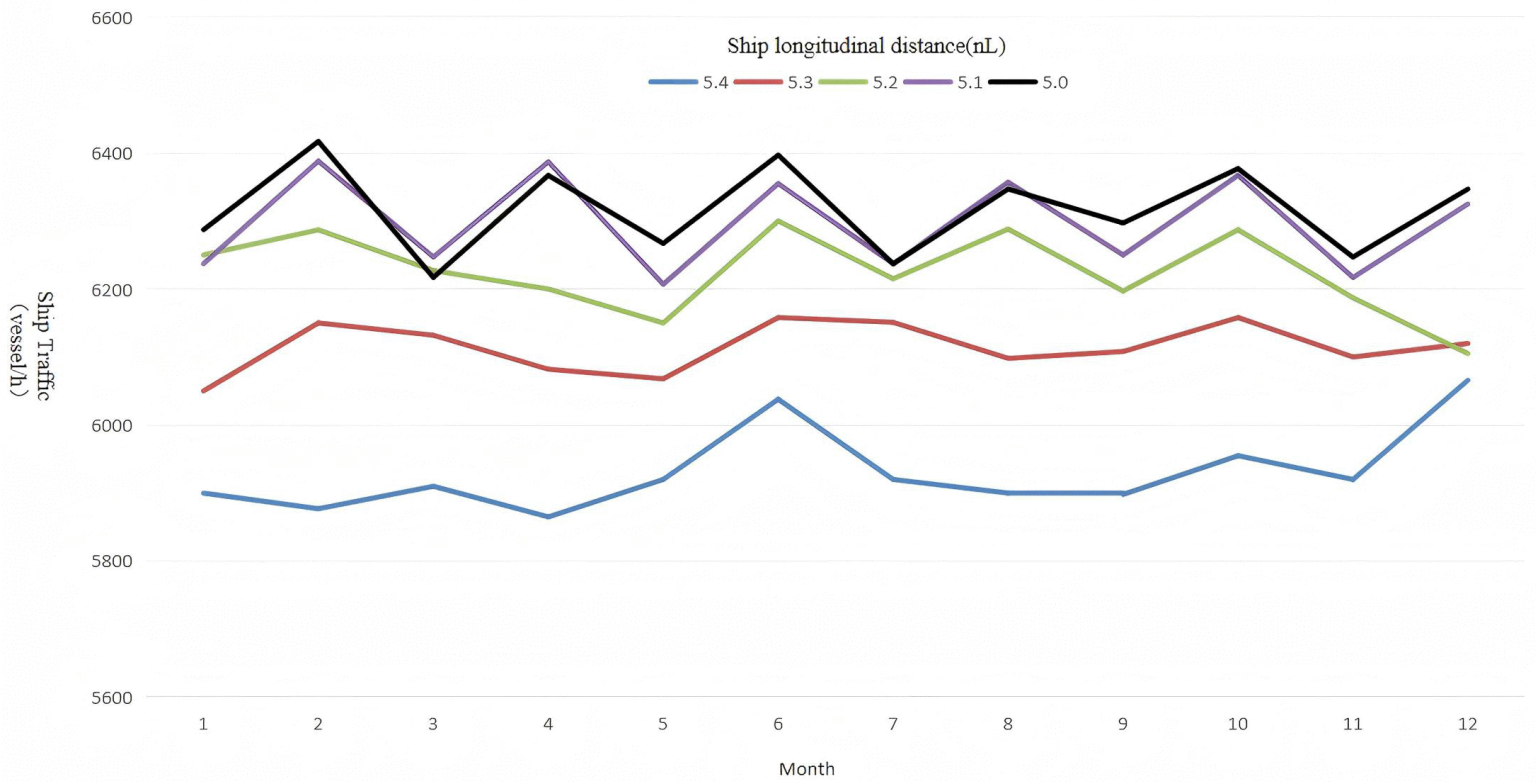
Passage capacity of eastbound vessels at different longitudinal spacing.

**Fig 17:**
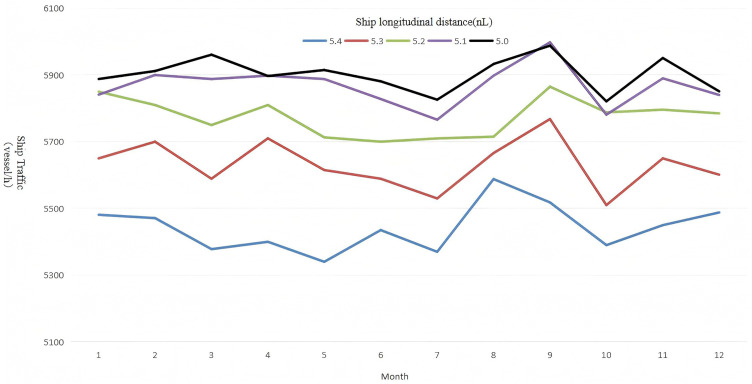
Passage capacity of westbound vessels at different longitudinal spacing.

To identify an appropriate longitudinal spacing for the Changshan Waterway, the baseline spacing used in the simulation model, set at 5.5 times the vessel length (5.5L), was gradually reduced to 5.4L, 5.3L, 5.2L, 5.1L, and 5.0L. Simulation experiments were conducted under each spacing condition to evaluate the resulting changes in throughput capacity. The corresponding simulation outputs are presented in Figs 16 and 17.

The results indicate that moderate reductions in longitudinal spacing significantly increase vessel density within the channel. As shown in Fig 17, for both eastbound and westbound traffic, throughput capacity increases as longitudinal spacing decreases, although the rate of increase gradually diminishes. When the spacing reaches 5.0L, the resulting throughput capacity becomes very close to that obtained at 5.1L, indicating that a critical spacing threshold has been reached. Further reductions in spacing beyond this point lead to a decline in throughput capacity.

At a longitudinal spacing of 5.0L, the throughput capacity of the eastbound channel reaches 75,810 vessels per year, representing an increase of 8.09 percent compared with the baseline capacity of 70,132 vessels per year at a spacing of 5.5L. Similarly, the westbound channel achieves a throughput capacity of 70,814 vessels per year, which is 8.53 percent higher than the baseline value of 65,248 vessels per year under the same initial spacing condition.

These findings suggest that reducing longitudinal spacing within an appropriate safety range can improve navigation efficiency and enhance channel throughput capacity. Based on the simulation results, it is recommended that longitudinal vessel spacing in the Changshan Waterway be controlled within the range of 5.0L to 5.5L. This range provides a balanced trade-off between operational safety and traffic efficiency under current navigation conditions.

## 4. Conclusions

This study presents an integrated AIS-driven microsimulation framework for throughput capacity assessment in constrained waterways, demonstrated through a case study of the Changshan Waterway.

Based on vessel AIS trajectory data from March 2024, key traffic flow characteristics were extracted and calibrated using the L-M optimization algorithm. The results show that vessel arrival processes follow a Poisson distribution, while vessel length and operating speed are well described by Gaussian distributions. Spatial analysis also quantifies the typical longitudinal safety spacing maintained between consecutive vessels. Using the calibrated traffic characteristics, a microscopic simulation model was established in AnyLogic, incorporating the traffic separation scheme and geometric constraints of the waterway. The model was validated against observed traffic data, showing average errors below 7% for both directions. By progressively increasing traffic demand, the practical throughput capacity of the Changshan Waterway was estimated. The current saturation level is 66.4%, which falls within service Level C but is approaching the critical threshold of 70%. Sensitivity analyses indicate that adjusting vessel speeds to the range of 10–11 knots and controlling longitudinal spacing within 5.0–5.5 times the vessel length can further enhance throughput capacity by approximately 6–9%.

Overall, the proposed framework demonstrates practical applicability for capacity assessment in constrained, traffic-intensive waterways. The findings provide quantitative references for throughput evaluation and traffic management in waterways with similar navigational characteristics.
